# Endogenous hydrogen peroxide increases biofilm formation by inducing exopolysaccharide production in *Acinetobacter oleivorans* DR1

**DOI:** 10.1038/srep21121

**Published:** 2016-02-17

**Authors:** In-Ae Jang, Jisun Kim, Woojun Park

**Affiliations:** 1 Laboratory of Molecular Environmental Microbiology, Department of Environmental Science and Ecological Engineering, Korea University, Seoul 02841, Republic of Korea

## Abstract

In this study, we investigated differentially expressed proteins in *Acinetobacter oleivorans* cells during planktonic and biofilm growth by using 2-dimensional gel electrophoresis combined with matrix-assisted laser desorption time-of-flight mass spectrometry. We focused on the role of oxidative stress resistance during biofilm formation using mutants defective in alkyl hydroperoxide reductase (AhpC) because its production in aged biofilms was enhanced compared to that in planktonic cells. Results obtained using an *ahpC* promoter-*gfp* reporter vector showed that aged biofilms expressed higher *ahpC* levels than planktonic cells at 48 h. However, at 24 h, *ahpC* expression was higher in planktonic cells than in biofilms. Deletion of *ahpC* led to a severe growth defect in rich media that was not observed in minimal media and promoted early biofilm formation through increased production of exopolysaccharide (EPS) and EPS gene expression. Increased endogenous H_2_O_2_ production in the *ahpC* mutant in rich media enhanced biofilm formation, and this enhancement was not observed in the presence of antioxidants. Exogenous addition of H_2_O_2_ promoted biofilm formation in wild type cells, which suggested that biofilm development is linked to defense against H_2_O_2_. Collectively, our data showed that EPS production caused by H_2_O_2_ stress enhances biofilm formation in *A. oleivorans*.

Many bacterial species exist in multicellular communities known as biofilms that can attach to any biotic or abiotic surface and at the interface between air and water in many different habitats^1^. The characteristics of cells within biofilms differ from those of planktonic cells. Among the biofilm-specific traits are antibiotic tolerance and resistance, slow growth, metabolic shift, production of extracellular matrix (ECM), and different cellular surface properties^2^. These physiological and phenotypical transitions result from the fine-tuned regulation of biofilm-specific genes involved in quorum sensing, the expression of small RNAs, cyclic diguanosine-5′-monophosphate (c-di-GMP) signaling, flagella biosynthesis, and ECM production^2^,^3^,^4^. The ECM is composed of exopolysaccharides, nucleic acids, and proteins, which surround biofilms and increases their tolerance to environmental chemicals^5^,^6^. This matrix also helps bacterial pathogens avoid host immune responses^7^. High ECM production creates a barrier that limits the penetration of various chemicals, including oxygen, nutrients, wastes, and antibiotics^8^,^9^. Because cells occupy different locations within a biofilm, they encounter different concentrations of chemicals, leading to heterogeneity in gene expression and physiology^2^.

Nonpolar, diatomic oxygen gas is small and freely diffusible through biological membranes, which is an important characteristic for aerobic respiration, where it is used as an electron acceptor^10^. Reactive oxygen species (ROS) can be generated during either respiratory or fermentative cellular metabolism, mainly as an unavoidable consequence of electron transfer from autooxidizable flavoproteins (such as NADH dehydrogenase and fumarate reductase) to O_2_^10^,^11^. Most aerobic and microaerophilic organisms have evolved protective mechanisms against ROS stress^12^. Successive electron acceptance by O_2_ generates superoxide (O_2_^−^) and hydrogen peroxide (H_2_O_2_). H_2_O_2_ reacts with ferric iron, thereby forming highly toxic hydroxyl radicals (HO^•^) through the Fenton reaction^10^. In bacterial cells, superoxide is dismuted by superoxide dismutase (SOD), and H_2_O_2_ is targeted by catalases and alkylhydroperoxidase^10^. However, excessive ROS is toxic to almost all cellular processes because of damaging effects on proteins and DNA^13^. Many environmental chemicals such as redox dyes, antibiotics, and organic and metal pollutants are also sources of ROS production^14^. In bacteria, endogenous and exogenous ROS are sensed by many redox-active transcriptional factors, such as OxyR, SoxR, OhrR, and PerR, which turn on genes involved in removing ROS and defending against their deleterious effects^10^,^15^,^16^,^17^. In response to H_2_O_2_ exposure, OxyR tetramers are activated through the formation of intra-disulfide bonds between cysteine residues. Several OxyR-controlled genes, including peroxiredoxin (*ahpC*), catalase (*katG*), and thioredoxin reductase (*trxB*), are involved in H_2_O_2_ detoxification^14^. Alkyl hydroperoxidase, which is composed of peroxiredoxin (AhpC) and the cognate reductase (AhpF), also plays an important role in scavenging endogenous H_2_O_2_^18^.

However, little is known about the oxidative stress response in *Acinetobacter* species. Non-pathogenic *Acinetobacter* species and the pathogen *A. baumannii* can experience different levels of ROS stresses during growth in their natural habitats and hosts, respectively^19^,^20^. *A. oleivorans* DR1, a soil-derived bacterium, forms biofilms at oil-water interfaces and requires the ECM for protection under these oily conditions. ROS stress is generated in *A. oleivorans* DR1 during growth on alkanes^20^,^21^,^22^. However, the relationship between the ROS stress responses and biofilm formation has not been explored in *Acinetobacter*. In this study, we used a proteomic approach to identify proteins important for biofilm formation and maintenance. Our data showed that the level of OxyR-regulated AhpC remains high in aged biofilms and that deletion of the *ahpC* gene appears to promote early biofilm formation by inducing exopolysaccharide (EPS) production. Furthermore, the addition of exogenous H_2_O_2_ or antioxidants modulated biofilm formation by altering EPS production.

## Results

### Differentially expressed proteins in mature and aged biofilms

To identify proteins associated with biofilm formation and maintenance, we employed a proteomics-based approach using *A. oleivorans* DR1 as a model soil microorganism. Among the 49 and 60 proteins that were upregulated in 24-h mature (Table 1 and Supplementary Table S1) and aged 48-h (Table 2 and Supplementary Table S2) biofilms, 12 and 16 proteins, respectively, were chosen for further analysis because their expression differences were higher than 1.5-fold. The predicted functions of 12 upregulated proteins detected in mature biofilms are related to histidine metabolism (HutI), guanine and quinone biosynthesis (UbiE, GuaB), outer membrane receptors (OprC, FabA), acetoin oxidoreductase (AcoB), and tRNA-dihydrouridine synthase (DusA) as well as two unknown proteins. Sixteen proteins detected in aged biofilms are related to oxidative stress defense (AhpC, Gpx), electron transfer flavoprotein and ATP synthase (EtfA, AtpD, and NudF), dehydrogenases (Dho, GabD, and Mdh), outer membrane receptors (FepA), histidine metabolism (HutU), and pili biogenesis (PilH).

In addition, mature and aged biofilms shared 3 common proteins: fumarate hydratase (FumC), a putative alcohol dehydrogenase (Adh), and a TonB-dependent receptor-like protein (CirA). These 3 proteins found in both biofilms may be important for biofilm maintenance. Upregulation of both alkyl hydroperoxide reductase (AhpC) and glutathione peroxidase (Gpx) implies that their function, the prevention of ROS toxicity, is essential for the maintenance of aged biofilms.

### Transcriptional regulation of *ahpC* expression in biofilms

**Figure 1:**
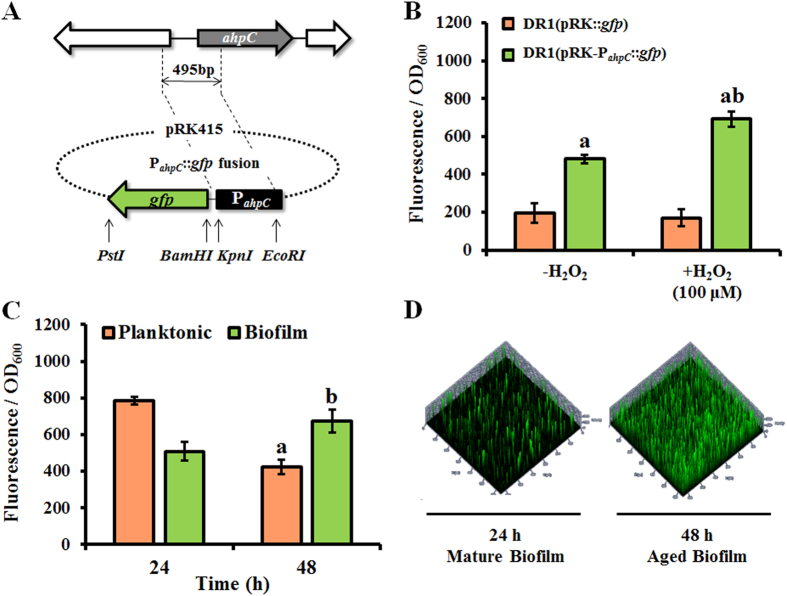
Analysis of *ahpC* gene expression. (**A**) Construction of the *ahpC* promoter *gfp* bioreporter. (**B**) Expression from the *ahpC* promoter by cells grown in LB medium with and without H_2_O_2_ (100 μM). (**C**) GFP fluorescence intensity of planktonic cells and biofilms grown in LB medium at 24 and 48 h. (**D**) CLSM images of the *ahpC::gfp* reporter strain in biofilms at 24 and 48 h. All samples were analyzed in triplicate. Statistical analyses were conducted using Student’s *t*-test. A letter on the bar graph indicates the level of significance.

Expression of *ahpC* was monitored in both planktonic and biofilm cells using the *A. oleivorans* DR1 strain, which contains a green fluorescence protein (*gfp*) reporter construct (pRK-P_*ahpC*_::*gfp*) (Fig. 1A). The involvement of OxyR in the regulation of *ahpC* gene expression was confirmed^23^. It has been reported that aerobic cells can release H_2_O_2_^24^, which was also observed in our reporter assay when cells were grown in rich media without added H_2_O_2_ (Fig. 1A,B). Our reporter construct responded to H_2_O_2_ (100 μM), as shown in Fig. 1A,B. In contrast to the 1.5-fold higher GFP expression observed in mature (24-h) planktonic cells versus biofilm cells at 24 h, 1.6-fold greater induction of *ahpC* expression was observed in aged biofilms compared to planktonic cells at 48 h (Fig. 1C), which is consistent with our proteomic data showing that AhpC protein levels remain high in aged biofilms. This differential GFP expression was visualized using confocal laser scanning microscopy (CLSM) (Fig. 1D). Based on these results, it is apparent that cells in growing planktonic culture in rich medium are under more oxidative stress at 24 h than at 48 h. Conversely, cells in aged biofilms appear to be under more oxidative stress than those in mature biofilms.

### Early biofilm formation in an *ahpC* deletion mutant

**Figure 2:**
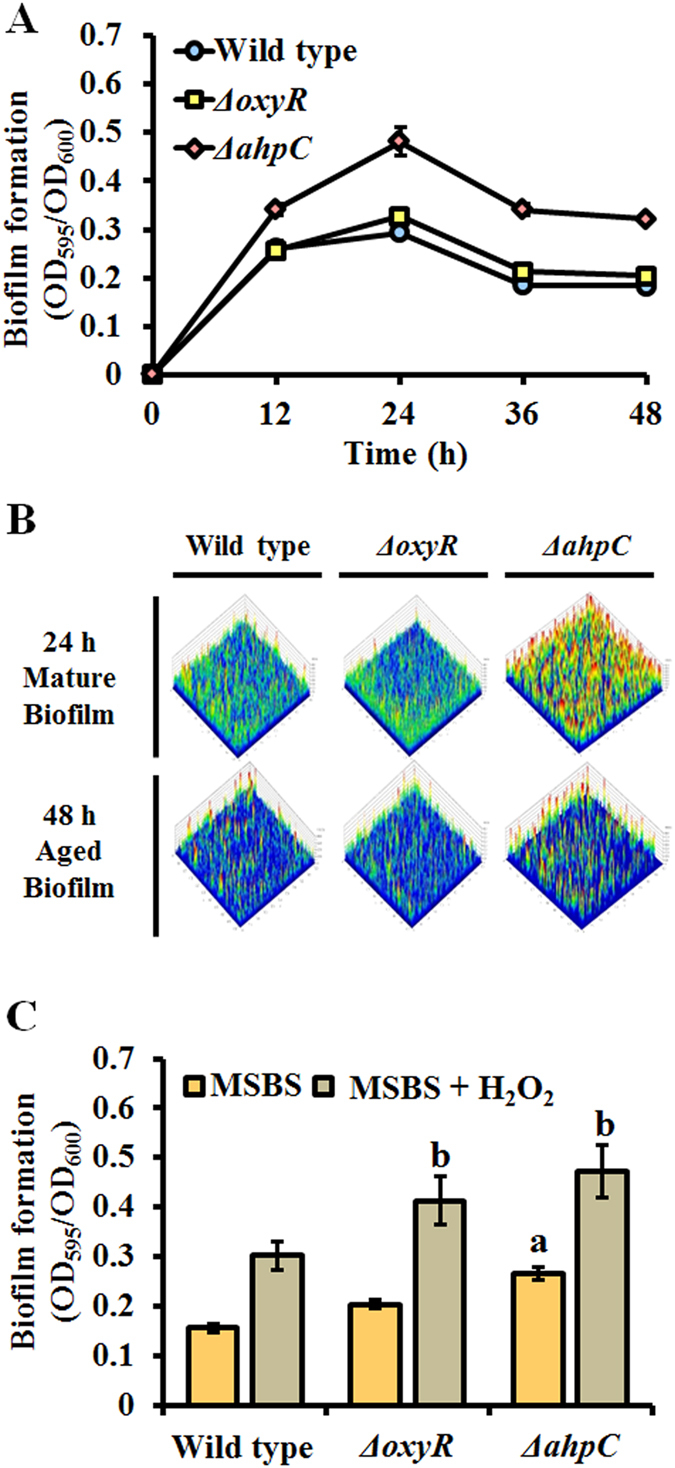
Quantification of biofilm formation under different conditions. (**A**) Biofilm formation was measured in 48-well microtiter plates containing LB medium at 30 °C. The OD_600_ was used to normalize the data. (**B**) Three-dimensional images of biofilm structures at 24 and 48 h, obtained using CLSM. The images were obtained after using the live/dead cell staining dyes SYTO9 and PI, respectively. (**C**) Biofilm formation was measured in MSBS medium containing 100 μM H_2_O_2_ in 48-well microtiter plates at 30 °C. The OD_600_ was used to normalize the data. All samples were analyzed in triplicate. Statistical analyses were conducted using Student’s *t*-test. A letter on the bar graph indicates the level of significance.

Deletion of *ahpC* or its regulator *oxyR* led to a slight growth defect in rich medium (LB) when compared to the growth of the wild type strain (Supplementary Fig. S1). Interestingly, the defect varied depending on the medium used. For wild type cells, optimal growth was observed in nutrient broth (NB), and severe growth defects were observed for both mutants when grown in this medium. However, when growth was monitored in another rich medium (LB medium) only a slight growth defect was observed. However, no growth defect was observed in MSB containing succinate (MSBS) medium. Thus, we used LB and MSBS medium to assess changes in the timing of biofilm formation. Despite the slight growth defect of the *ahpC* mutant, it exhibited 1.7-fold more mature biofilm formation at 24 h and 1.5-fold more aged biofilm at 48 h than the wild type (Fig. 2A). The dispersal rates of the *ahpC* mutant was similar to those of the wild type and *oxyR* mutant in LB media (Fig. 2A). The same conclusion was drawn using CLSM images stained with live/dead dye (Fig. 2B). However, although deletion of the master regulator gene *oxyR* resulted in a growth defect similar to that of the *ahpC* mutant, the timing of biofilm formation for this mutant did not differ from that of wild type cells (Fig. 2A). This discrepancy cannot be clearly explained; however, it is possible that other enzymes that detoxify H_2_O_2_ and modify cellular structures are upregulated in the *oxyR* mutant, which modulates biofilm formation, but not planktonic growth. Addition of exogenous H_2_O_2_ to MSBS promoted biofilm formation in all strains; 2-fold and 1.6-fold increased biofilm formations were observed with the wild type and *ahpC* mutant strains, respectively (Fig. 2C). Results obtained using the a*hpC* complementary strain verified our finding (Supplementary Fig. S2). Taken together, our data suggest that early biofilm formation in the *ahpC* mutant may be a defense mechanism to avoid oxidative stress.

### Enhanced EPS production in the *ahpC* mutant

**Figure 3:**
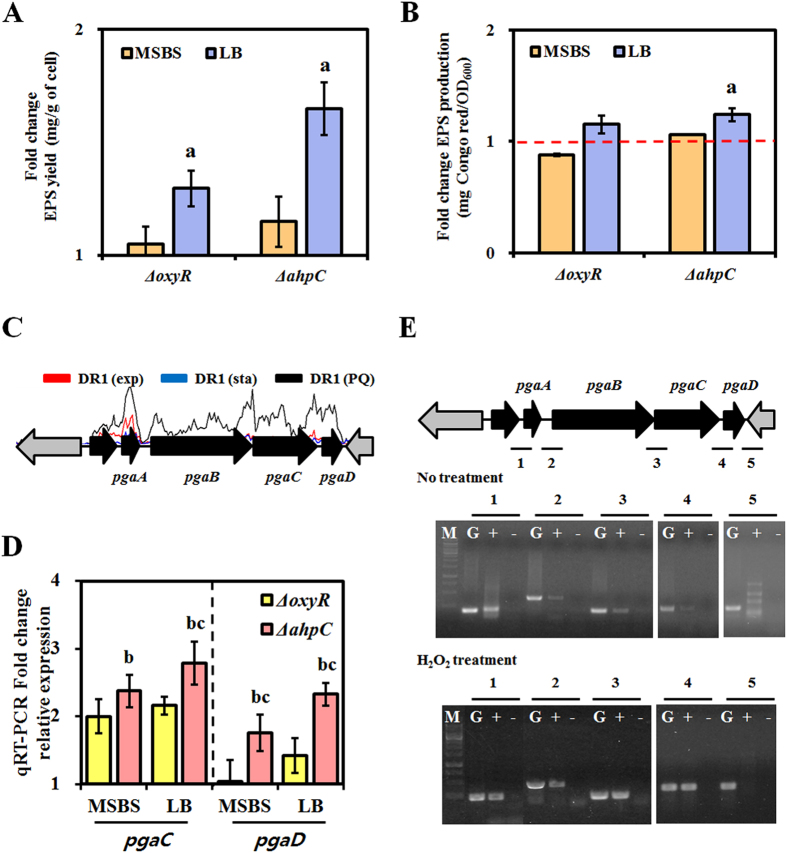
Measurement of EPS production. (**A**) Total EPS produced by cells grown in MSBS and LB media was divided by the weight of cells (in g) to normalize the data. Fold-changes in EPS production by the mutant strains compared to that produced by wild type strains were determined. (**B**) Quantification of EPS production by cells grown in MSBS and LB media using Congo red staining. (**C**) Analysis of PNAG operon expression, based on RNA-sequencing data. Strains were grown to exponential and stationary phase in NB medium. RNA-sequencing data from *A. oleivorans* DR1 grown in NB medium treated with 1 mM Paraquat. (**D**) Quantitative real-time PCR to determine the relative expression of PNAG. Relative expression of *pgaC* and *pgaD* (PNAG operon) in the *ahpC* and *oxyR* mutant strains was determined after growth in MSBS and LB media and compared to their expression levels observed in the wild type strain. (**E**) Semiquantitative real-time PCR to assess the expression level of genes in the PNAG operon in *A. oleivorans* DR1, with and without H_2_O_2_. Lines under the arrows indicate the PCR-amplified intergenic regions (**G**), cDNA (+), and the negative control (–[no reverse transcriptase added]). M, 1-kb DNA ladder. All samples were analyzed in triplicate. Statistical analyses were conducted using Student’s *t*-test. A letter on the bar graph indicates the level of significance.

**Figure 4:**
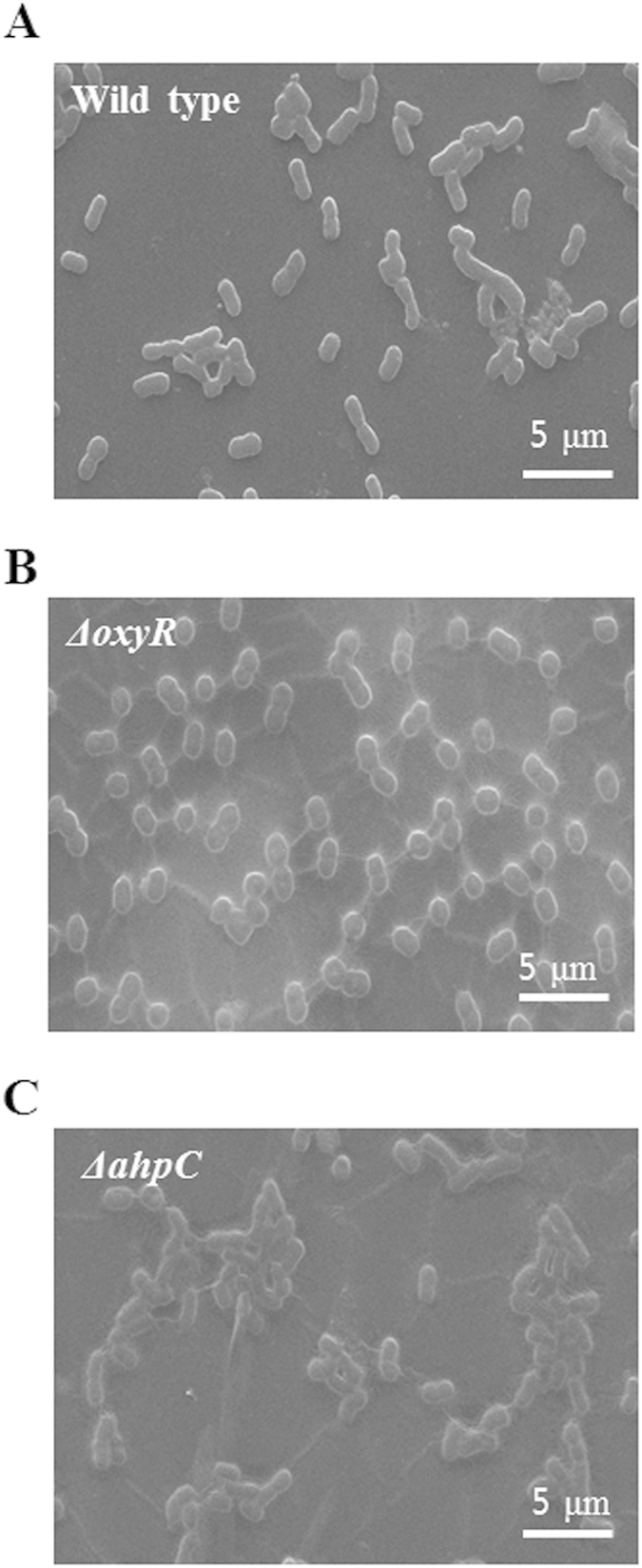
SEM analysis of EPS production in the *ahpC* mutant. SEM images of (**A**) wild type, (**B**) *oxyR* mutant, and (**C**) *ahpC* mutant biofilms incubated for 24 h were obtained at 3,000 × magnification. Partial extracellular matrix-like structures were observed. All biofilm samples were fixed, dried, and coated with platinum (Pt) before imaging. Scale bars = 5 μM and 1 μM in the 3,000 × magnification images, respectively.

Production levels of the major biofilm-contributing factor (EPS) were measured in the *ahpC* mutant, the *oxyR* mutant, and wild type cells (Fig. 3A). The *ahpC* mutant produced 1.7-fold more EPS than did the wild type strain when grown in LB medium, but not when grown in minimal MSBS medium. Based on this finding, together with the early biofilm formation observed with the *ahpC* mutant, a reasonable conclusion is that cells grown in rich media are under more oxidative stress than those grown in minimal medium, which might lead to higher EPS production. It is noteworthy that in our previous study, both NaCl and hexadecane stress induced EPS production in the wild type strain^25^. However, the molecular mechanism explaining the relationship between those stresses and EPS production has not been explored^25^. Congo red (CR) staining also showed slightly enhanced EPS production in the *ahpC* mutant (Fig. 3B). Very little has been done to characterize EPS production in *Acinetobacter* species. The genome of *A. oleivorans* DR1 contains 3 possible EPS operons: 2 poly-N-acetyl glucosamine (PNAG) operons (AOLE14655–AOLE14675 and AOLE06340–AOLE06360) and the K locus, which is known to encode an important EPS complex in *A. baumannii*^26^ (Supplementary Figs S3 and S4). The PNAG operon is composed of 4 genes, *pgaABCD*, which are required for the synthesis and transport of PNAG. Our previous RNA-seq data obtained under oxidative stress showed that only the first PNAG operon (AOLE14655–AOLE14675) was highly induced following Paraquat treatment (1 mM), suggesting that this EPS operon is likely controlled by oxidative stress (Fig. 3C). When cells were grown aerobically in MSBS, higher levels of *pgaC* and *pgaD* expression (2.3-fold and 1.7-fold higher, respectively), which encode the catalytic subunits of PNAG synthase, were observed in the *ahpC* mutant by quantitative reverse transcriptase PCR (qRT-PCR). Here, a higher fold-change increase in the expression of *pgaC* and *pgaD* was observed in the *ahpC* mutant (2.8-fold and 2.3-fold, respectively) when grown in LB medium (Fig. 3D). Consistent with our RNA-seq data, our semi-quantitative, real-time RT-PCR data showed that this PNAG gene cluster is indeed an operon that could be induced by H_2_O_2_ (Fig. 3E). Higher RT-PCR band intensities were noted in cells exposed to H_2_O_2_, and higher EPS production in biofilms was also observed by scanning electron microscopy (Fig. 4). In addition, *oxyR* and *ahpC* mutant cells growing exponentially on cover glass showed EPS-like extracellular structures.

### Increased H_2_O_2_ production in the *ahpC* mutant

**Figure 5:**
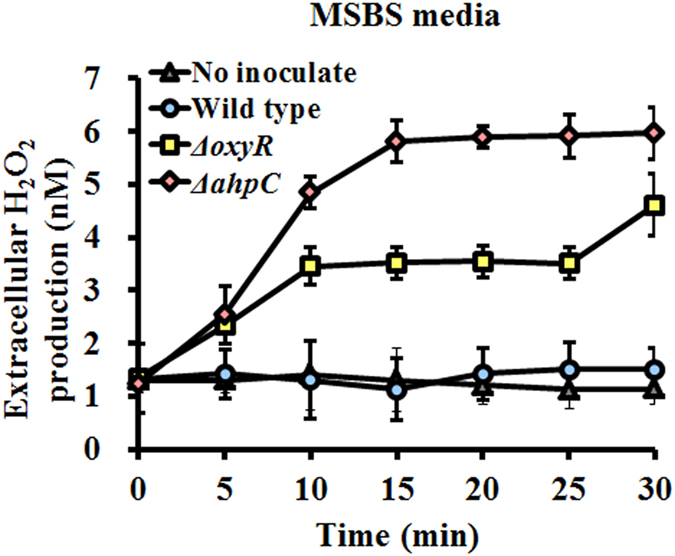
Detection of H_2_O_2_ generation. H_2_O_2_ generation during cell growth in early exponential phase of WT and Δ*oxyR* and Δ*ahpC* mutants for 30 min in minimal media.

**Figure 6:**
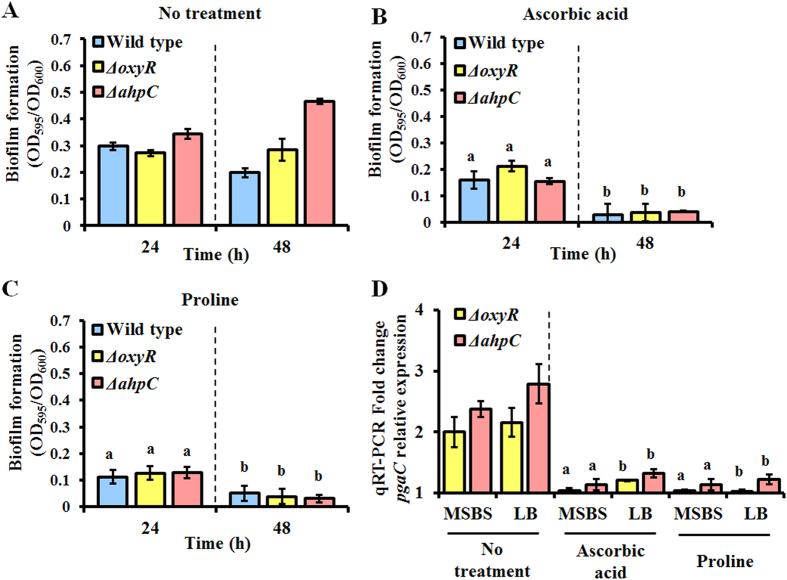
Biofilm formation under antioxidants-amended conditions. (**A**) Biofilm formation without antioxidants. (**B**) Biofilm formation in cultures treated with 1/2× the MIC of ascorbic acid. The MIC of ascorbic acid was determined to be 256 μg/ml. (**C**) Biofilm formation in cultures treated with 1/2× the MIC of proline. The MIC of proline was determined to be 128 μg/ml. (**D**) Relative expression of *pgaC* in the *ahpC* and *oxyR* mutants was determined in MSBS or LB medium, with either ascorbic acid or proline production being compared to the corresponding expression level in the wild type strain. All samples were analyzed in triplicate. Statistical analyses were conducted using Student’s *t*-test. A letter on the bar graph indicates the level of significance.

Extracellular H_2_O_2_ accumulated in both the *oxyR* and *ahpC* mutants grown in MSBS at early exponential phase due to a defect in H_2_O_2_ removal during aerobic growth (Fig. 5). Much higher H_2_O_2_ production (3.9-fold increased within 30 min) was noted with the *ahpC* mutant, and results obtained using the *ahpC* complementary strain verified this finding (Supplementary Fig. S5). H_2_O_2_ production could not be measured in rich media, such as NB or LB, owing to interference from the nutrient components. Interestingly, when well-known antioxidants, such as ascorbic acid or proline, were added^27^,^28^, biofilm formation by both the wild type and mutant strains appeared to decrease at 24 h (Fig. 6A–C). Moreover, *pgaC* expression was decreased in all strains when mutants were grown aerobically in MSBS or LB medium with antioxidants such as ascorbic acid or proline (Fig. 6D). Both wild type and mutants appeared to have similar levels of *pgaC* expression under antioxidants-amended conditions.

## Discussion

Our initial rationale for this proteomic study was to identify important biofilm-related proteins in *Acinetobacter* species, which have been poorly explored compared to other important environmental microbes. However, only a small number of differentially expressed proteins could be characterized in our study due to the low resolution of our proteomic analysis. In fact, many proteins previously linked to biofilm formation and maintenance were not found, including those related to stress responses, motility, curli production, exopolysaccharide production, c-di-GMP synthesis and degradation, as well as other well-known contributors to biofilm build up^29^,^30^,^31^. These data suggested that the abovementioned proteins may be regulated in a more sophisticated manner, such that the 2 time points used and/or the low number of proteins identified were not sufficient to detect these biofilm-specific proteins. Only a few proteins that may be related to biofilm formation and maintenance were found (Tables 1 and 2 and Supplementary Tables S1 and S2). Among these were imidazolonepropionase (HutI) and urocanate hydratase (HutU), two enzymes involved in the metabolism of histidine, which can be converted to molecules related to DNA synthesis, such as purines and pyrimidines. DNA synthesis is linked to extracellular DNA generation for biofilm formation^32^. A hypothetical protein encoded by AOLE_07010 was 260-fold upregulated in our mature biofilm (Table 1). Bioinformatics analysis suggested that this protein shares 35% protein identity with a lysophospholipase from *Legionella pneumophila*^33^. The role of this protein in biofilm formation is unclear. Outer membrane protein C (OprC, encoded by AOLE_18500) was upregulated 10.6-fold in mature biofilms. Our previous data suggested that *oprC* is an OxyR-controlled gene^23^ and that EtfA (encoded by AOLE_04180), which is involved in electron transport, and FumC (encoded by AOLE_07235) are highly upregulated by H_2_O_2_ and superoxide generators, respectively. OxyR is a master regulator of oxidative stress defense against H_2_O_2_ in many bacterial species, and in *Escherichia coli*, its regulon includes *ahpCF, katG, gorA, trxC*, and *dps*^34^,^35^. Interestingly, the peroxiredoxin AhpC was upregulated in aged biofilms only (Table 2). AhpC is known to be very abundant in bacterial cells throughout growth^36^. The higher level of AhpC detected in aged biofilms at 48 h shows its importance in maintaining biofilm structure and defense against ROS stress in aged biofilms (Table 2 and Fig. 1). Our proteomic data indicated that these ROS defense-related proteins might play an important role in the maintenance of *A. oleivorans* biofilms.

A recently emerging topic in biofilm research is biofilm structure-driven physiological differentiation resulting from gradients of oxygen, nutrients, waste products, and signaling molecules^11^. The oxidative stress response has been reported to be induced in cells within biofilms to reduce ROS stress encountered during growth^37^,^38^,^39^. Oxidative stress generated by endogenous ROS in biofilm cells is also known to enhance bacterial diversity and alter bacterial physiology^37^,^40^. For example, nutritional starvation in *Pseudomonas aeruginosa* biofilms confers increased antibiotic resistance through stringent signaling, and inactivation of the stringent response significantly impaired catalase and superoxide dismutase activity^41^. Our previously study also showed that protein expression of Cu, Zn-superoxide dismutase (SodC) and thiol peroxidase (Tpx) is increased in *E. coli* O157:H7 biofilms, and mutation of s*odC* and *tpx* impaired biofilm formation^42^. Iron stimulates the formation of rugose biofilms of *E. coli*, and induction of superoxide stress through the addition of a superoxide generator or mutation of *sodA* and *sodB* promotes the development of rugose biofilms^43^.

Few studies have examined the relationship between bacterial EPS production and ROS stress. Here, we provide evidence showing that a PNAG operon is upregulated in wild type cells by the addition of exogenous H_2_O_2_ and is upregulated in *ahpC* mutant cells even without exogenous H_2_O_2_ (Figs 3 and 4), which resulted in greater biofilm formation in the *ahpC* mutant (Fig. 2). Many microorganisms, such as *E. coli* and *A. baumannii*, possess a PNAG operon comprised of *pgaABCD*^44^,^45^ (Supplementary Fig. S3). We used the PNAG operons of *E. coli* O157:H7 and *A. baumannii* in a bioinformatics analysis to reveal the EPS operons in the genome of *A. oleivorans* DR1. Two PNAG operons were identified in *A. oleivorans* DR1 using a BLASTP search of the NCBI database. One of the operons spans nucleotides 3, 132, 165–3, 137, 139 and the other spans nucleotides 1, 382, 343–1, 389, 531. The loci were annotated as AOLE_14655–AOLE_14675 and AOLE_06340–AOLE_06360. In this study, only the former loci were induced by ROS stress (Fig. 3).

In *E. coli*, the *pgaA* gene product contains a predicted porin (807 aa) domain and functions as an outer membrane protein that facilitates PNAG translocation and retains PNAG on the cell surface. The predicted PgaA protein encoded in the first PNAG operon (PNAG1) in *A. oleivorans* DR1 and *A. baumannii* shared 96% identity. However, *E. coli pgaA* was not related to the *pgaA* genes of *Acinetobacter* species, which are shorter than that in *E. coli*. Moreover, an unknown gene located upstream of *pgaA*, which is annotated as a YaeQ family protein, is absent from the genome of *E. coli.* The PNAG2 operon also contains a *pgaA* gene encoding a biofilm synthesis-related protein (812 aa) that shares 25% and 83% amino acid identity with *E. coli* and *A. baumannii pgaA*, respectively. In the PNAG1 operon, *pgaB* is annotated as a PGA synthesis lipoprotein precursor functioning in PNAG export (664 aa), which shares 38% and 32% identity with *E. coli* and *A. baumannii pgaB*, respectively. *PgaC* is annotated as an N-glycosyltransferase (418 aa), and it shares 52% identity with both *E. coli* and *A. baumannii pgaC*. In addition, several amino acids important for the catalytic activity of glycosyltransferases are conserved in PgaC1 (Asp^141^, Asp^234^, Gln^270^, Arg^273^, and Trp^274^) and PgaC2 (Asp^135^, Asp^228^, Gln^264^, Arg^267^, and Trp^268^), as shown in Supplementary Fig. S6 46. The function of the *pgaD* gene product is unknown; however, in *E. coli,* it has been predicted to localize to the cytoplasm and assist the glycosyltransferase PgaC^44^,^45^. *A. baumannii* also contains a K locus that is involved in the production of complex polysaccharides^26^. Using BLASTP, a K locus, including 19 genes, was identified in the genome of *A. oleivorans* DR1. (Supplementary Fig. S4). Some of the genes in this locus were upregulated under oxidative stress in our RNA-seq data (Spplementary Fig. S4). Further characterization of the EPS operons under different growth conditions is needed to clarify their involvement in biofilm formation.

H_2_O_2_ production, biofilm formation, and EPS expression were measured in an *A. oleivorans* DR1 *soxR* mutant and *E. coli* MG1655 *katG*, Hpx (*katG, katE*, and *ahpCF*)^24^,^47^, as shown in Supplementary Fig. S7. Interestingly, all mutants appeared to show greater biofilm formation by inducing several EPS synthesis genes^48^. This additional information supported our findings, indicating that EPS production caused by oxidative stress increased biofilm formation and genes involved in EPS production were upregulated under oxidative stress conditions.

In summary, our data provide evidence that AhpC affects biofilm formation. Exogenously added and endogenously produced H_*2*_O_2_ enhanced early biofilm formation in the wild type and *ahpC* mutant strains, respectively. Early biofilm formation appears to be caused by induction of the PNAG operon, which was supported by the results of several assays.

## Methods

### Bacterial strains, culture conditions, and RNA-seq

The bacterial strains that were used in this study are shown in Supplementary Table S3. We used a diesel-degrading *A. oleivorans* strain DR1 that was isolated from the soil in a Korea University paddy field (Deokso, Gyeonggi-Do, Korea). Its genome was completely sequenced in a previous study^21^. *A. oleivorans* DR1 was grown at 30 °C in NB with aeration by shaking. When required, kanamycin (50 μg/ml) was added to the medium. Growth was monitored by measuring the optical density of the cultures at 600 nm (OD_600_) using a biophotometer (Eppendorf, Hamburg, Germany). The complete genome sequence of strain DR1 can be found in GenBank (accession no. CP002080). The genome sequence and annotation information for *A. oleivorans* DR1 was obtained from the National Center for Biotechnology Information (NCBI) database (accession number NC_014259.1). Mapped reads were visualized using BamView and Artemis software. The RNA-seq data were deposited in the NCBI Gene Expression Omnibus site under accession number GSE44347 (exponential and stationary phase grown in NB medium) and GSE70356 (1 mM PQ treatment).

### Construction of the *ahpC* complementation plasmid

The *ahpC* gene was amplified by PCR from genomic DNA and cloned into the pRK415 plasmid. The amplified fragment was digested by *Bam*HI and *Kpn*I, and then ligated into the *BamHI*/*KpnI* sites of pRK415, generating the pPK415-*ahpC* complementation plasmid. The sequence of the inserted fragment was confirmed using the M13_FP and *ahpC*_comple_F primers and the construct was inserted in *ahpC* mutant

### Two-dimensional gel electrophoresis (2-DE) proteomic analysis

Biofilms were collected for protein profiling, and each sample was analyzed in duplicate by 2-DE as previously described^46^. Cell pellets were sonicated for 10 s in lysis buffer (7 M urea, 2 M thiourea, 4% (w/v) 3-[(3-cholamidopropyl) dimethylammonio]-1-propanesulfonate (CHAPS), 1% (w/v) dithiothreitol (DTT), 2% (v/v) pharmalyte, and 1 mM benzamidine), using a Sonopuls sonicator (Bandelin). Proteins were extracted for 1 h at room temperature with vortexing. After centrifugation at 15,000 × *g* for 1 h at 15 °C, the insoluble material was discarded, and the soluble fraction was used for the 2-DE analysis. Protein loading was normalized by measuring protein concentrations by the Bradford assay. Immobilized pH gradient dry strips were equilibrated for 12–16 h with buffer containing 7 M urea, 2 M thiourea, 2% CHAPS, 1% DTT, and 1% pharmalyte, after which 800 μg of each sample was loaded. Isoelectric focusing (IEF) was performed at 20 °C using a Multiphor II electrophoresis unit and an EPS 3500 XL power supply (Amersham Biosciences), according to the manufacturer’s instructions. For IEF, the voltage was linearly increased from 150–3,500 V over 3 h for sample entry, followed by maintenance at 3,500 V, and focusing was complete after 96 kV•h. Prior to the second-dimension separation, the strips were incubated for 10 min in equilibration buffer (50 mM Tris-Cl, pH 6.8, containing 6 M urea, 2% SDS, and 30% glycerol), first with 1% DTT, then a second time with 2.5% iodoacetamide. The equilibrated strips were placed onto SDS-polyacrylamide gels (20 × 24 cm, 10%–16%), and electrophoresis was performed using a Hoefer DALT 2D system (Amersham Biosciences), according to the manufacturer’s instructions. The 2-DE gels were run at 20 °C for 1,700 Vh and then stained with Coomassie G250 and SYPRO^®^ Ruby gel stain. After electrophoresis, the gel was placed into a clean container and agitated on an orbital shaker with 400 ml of fixative solution (40% ethanol and 10% acetic acid) for 600 min. After fixing, the gel was hydrated with 400 ml of rehydration solution (5% ethanol and 5% acetic acid) for 30 min. Rehydration was performed 3 times with fresh rehydration solution. SYPRO^®^ Ruby gel stain (400 ml) was added, and the gel was agitated on an orbital shaker for 2 h. The gel was then transferred to a clean container and washed with 400 ml of wash solution for 30 min. Fluorescence images were acquired with a DIVERSITY CCD camera (Syngene) using a Cy3 emission filter for a 20-s exposure. Quantitative analysis of digitized images was performed using PDQuest software, version 7.0 (Bio-Rad), according to the manufacturer’s protocol. The quantity of each spot was normalized against the total valid spot intensity. Protein spots were selected when the variation in intensity deviated more 1.5-fold from that of the control or normal sample.

### Protein identification by matrix-assisted laser desorption time-of-flight mass spectrometry (MALDI-TOF/MS)

To identify proteins by peptide mass fingerprinting, spots were excised, digested with trypsin (Promega), mixed with α-cyano-4-hydroxycinnamic acid in 50% acetonitrile/0.1% TFA, and subjected to MALDI-TOF MS analysis (Microflex LRF 20; Bruker Daltonics). Spectra were collected from 300 shots per spectrum over an m/z range of 600–3,000 and were calibrated by 2-point internal calibration using trypsin auto-digestion peaks (m/z 842.5099, 2211.1046). The peak list was generated using Flex Analysis software, version 3.0. The thresholds used for peak picking were as follows: 500 for the minimum resolution of monoisotopic mass and 5 for the signal to noise ratio. The search program MASCOT, developed by Matrix Science, Inc. (http://www.matrixscience.com), was used for protein identification by peptide mass fingerprinting. The following parameters were used for the database search: trypsin as the cleaving enzyme, a maximum of 1 missed cleavage, iodoacetamide (Cys) as a complete modification, oxidation (Met) as a partial modification, monoisotopic masses, and a mass tolerance of ±0.1 Da. The PMF acceptance criteria were based on probability scoring.

### Biofilm assay

Overnight cultures of *A. oleivorans* DR1 were washed twice with PBS and inoculated at 10^6^ CFU/ml into fresh LB or MSBS medium in PVC 48-well microtiter plates (BD Biosciences). The plates were incubated at 30 °C without agitation for either 24 or 48 h. After incubation, the microtiter plates were rinsed with sterile water, and then 0.1% (w/v) crystal violet (CV) solution was added to stain the attached cells. After staining, the CV was removed, and the wells were rinsed with sterile water. The dye was dissolved in 95% ethanol, and the absorbance of the solubilized dye at 595 nm was then determined.

### CLSM experiments

Biofilm cells were stained with RedoxSensor Green (Invitrogen) for 15 min at room temperature and observed by CLSM (LSM700; Carl Zeiss, Jena, Germany). Confocal images of RedoxSensor Green-stained biofilm cells were observed under green fluorescent light (excitation wavelength: 490 nm, emission wavelength: 520 nm) to evaluate the height and density of the biofilms (C-Apochromat 403/1.20 W Korr M27; Carl Zeiss).

### EPS assay

EPS was isolated as described previously^46^. *A. oleivorans* DR1 wild type, *oxyR* mutant, and *ahpC* mutant strains grown under different conditions were centrifuged for 30 min at 12,000 × *g* and 4 °C. The supernatants were mixed with 3 volumes of cold ethanol and incubated overnight at 4 °C. After centrifugation (5,000 × *g*, 15 min, 4 °C), the pellets were suspended in 80% ethanol and centrifuged, and then washed 3 times. Then, the extracted EPS was freeze-dried and weighed.

EPS production was quantitatively measured based on CR binding, as described previously^49^. One milliliter of an overnight culture was washed with 1 ml of T-broth, and the cell density was determined by measuring the OD_600_. Bacterial suspensions in T-broth were resuspended in 1 ml of 0.004% (w/v) CR and incubated for 2 mg/ml at 30 °C with vigorous shaking. After 2 h, the cells were removed by centrifugation. The amount of CR remaining in the supernatant was determined by measuring the OD_490_ of the solution.

### Analysis of PNAG expression by qRT-PCR

For qRT-PCR, total RNA was isolated from 5 ml of exponentially growing cells (OD_600_ ~0.9) using the RNeasy Mini Kit (Qiagen), according to the manufacturer’s instructions. Total RNA (10 μg) was treated with DNase I at 37 °C for 1 h. Then, cDNA was synthesized from the DNase-treated total RNA to obtain first-strand cDNA suitable for PCR amplification using the RevertAid H Minus First Strand cDNA Synthesis Kit (Fermentas) and the primer pair AOLE_14655 F/R. qRT-PCR was performed using the iCycler iQ Real-Time PCR Detection System (Bio-Rad). cDNA was produced from the same RNA used for RT-PCR. For real-time RT-PCR, we used 1 μl of template cDNA, 5 pmol of primers, 0.5 × SYBR Green, and 1 U of Taq polymerase (Fermentas). Fluorescence was measured at the end of each 72 °C extension step and analyzed with iCycler iQ software (version 3.0). Melting curve analysis (60–95 °C in 0.5 °C increments) was performed to ensure PCR specificity. For quantification, the 16S rRNA gene was used as shown in Supplementary Table S4.

### H_2_O_2_ measurement

A horseradish peroxidase (HRP)-based assay using Amplex Red (AR) was employed to measure H_2_O_2_, as described previously^34^. Fluorescence was then measured using a fluorometer and converted to the H_2_O_2_ concentration using a standard curve. Note that a small amount of H_2_O_2_ was generated by the AR/HRP detection system itself; this amount was accounted for by the standard curve.

### Scanning electron microscopy of biofilms

Biofilm samples were grown on glass slides for 24 h. Then, the glass slides were rinsed once with PBS, immersed in half-strength Karnovsky’s solution (10% paraformaldehyde, 50% glutaraldehyde, 0.1 M sodium cacodylate buffer, and 200 mg/ml calcium chloride, pH 7.4), and incubated overnight. The glass slides were post-fixed with a 2% osmium tetroxide solution for 2 h. The fixed specimens were dehydrated in a graded series of ethanol (30%, 50%, 70%, 80%, 90%, and 100% [v/v]). The samples were then dried and sputter-coated with platinum using a plasma multicoater. All samples were examined with a scanning electron microscope (Quanta 240 FEG; FEI, Germany).

## Additional Information

**How to cite this article**: Jang, I.-A. *et al*. Endogenous hydrogen peroxide increases biofilm formation by inducing exopolysaccharide production in *Acinetobacter oleivorans* DR1. *Sci. Rep.* **6**, 21121; doi: 10.1038/srep21121 (2016).

## Supplementary Material

Supplementary Information

## Figures and Tables

**Table 1:** Proteins indicating different expression levels in 24-h mature biofilms and planktonic cells.

Spot no.	MW	pI	gene	Protein	Locus tag	Fold-^change^
7707	65.3	6.2		hypothetical protein	AOLE_07010	H^a^
7409	51.8	5.9	*hutI*	imidazolonepropionase	AOLE_00370	21.0
7806	94.1	6.0	*oprC*	outer membrane receptor protein, mostly Fe transport	AOLE_18500	10.6
8212	35.5	6.9	*dusA*	tRNA-dihydrouridine synthase A	AOLE_16645	10.1
7603	59.6	5.8	*fumC*	fumarate hydratase	AOLE_07235	7.7
9302	41.9	7.7	*ubiE*	S-adenosylmethionine: 2-DMK methyltransferase and 2-octaprenyl-6-methoxy-1,4-benzoquinone methylase	AOLE_17670	4.1
7510	52.3	6.0	*adh*	putative alcohol dehydrogenase	AOLE_06670	3.7
9601	62.8	7.5	*guaB*	inosine 5′-monophosphate dehydrogenase	AOLE_00780	3.6
7802	106.3	5.6	*fepA*	outer membrane receptor FepA	AOLE_14435	3.3
5403	48.2	5.1	*acoB*	acetoin:2,6-dichlorophenolindophenol oxidoreductase subunit beta	AOLE_10255	2.7
3806	101.1	4.8	*cirA*	TonB dependent receptor family protein	AOLE_02770	2.3
2309	40.8	4.6		hypothetical protein	AOLE_17675	1.8

^a^‘H’ indicates a protein that was exclusively detected in biofilm cells.

**Table 2:** Proteins indicating different expression levels in 48-h aged biofilms and planktonic cells.

Spot no.	MW	pI	gene	Protein	Locus tag	Fold-change
102	27.8	4.4	*ahpC*	peroxiredoxin	AOLE_13380	H^a^
8202	34.7	6.8	*dho*	dehydrogenase	AOLE_09905	H
204	37.9	4.3	*etfA*	electron transfer flavoprotein subunit alpha	AOLE_04180	H
5605	62.2	5.5	*gabD*	NADP-dependent aldehyde dehydrogenase	AOLE_06655	16.2
1804	92.2	4.7	*fepA*	TonB-dependent receptor	AOLE_09880	9.4
1904	104	4.7	*cirA*	TonB dependent receptor family protein	AOLE_02770	8.7
3304	42.9	5.1	*mdh*	malate dehydrogenase	AOLE_02365	2.7
4008	22.9	5.2	*nudF*	ADP-ribose pyrophosphatase	AOLE_04965	2.5
5709	74.5	5.4	*hutU*	urocanate hydratase	AOLE_00355	2.3
2005	16	4.9	*pilH*	protein pilH	AOLE_03150	2.1
2606	60.8	4.9	*atpD*	F0F1 ATP synthase subunit beta	AOLE_18565	1.9
6410	51.6	5.9	*adh*	putative alcohol dehydrogenase	AOLE_06670	1.9
1101	28.9	4.6	*gpx*	glutathione peroxidase	AOLE_18550	1.8
205	38.3	4.5	*etfA*	electron transfer flavoprotein subunit alpha	AOLE_04180	1.7
1006	16.2	4.7		hypothetical protein	AOLE_15935	1.6
6504	59.3	5.7	*fumC*	fumarate hydratase	AOLE_07235	1.5

^a^‘H’ indicates proteins that were exclusively detected in biofilm cells.
