# Supplementary material for: Endogenous hydrogen peroxide increases biofilm formation by inducing exopolysaccharide production in Acinetobacter oleivorans DR1
Source: Sci Rep. 2016 Feb 17;6:21121. doi: 10.1038/srep21121 (PMC4756669; doi:10.1038/srep21121)
Supplement: Supplementary Information [file srep21121-s1.pdf]

Supplementary Materials

Scientific Reports

Endogenous hydrogen peroxide increases biofilm formation by inducing exopolysaccharide production in *Acinetobacter oleivorans* DR1

In-Ae Jang, Jisun Kim and Woojun Park*

Laboratory of Molecular Environmental Microbiology, Department of Environmental Science and Ecological Engineering, Korea University, Seoul 02841, Republic of Korea

Running title: H_2O_2-induced biofilm formation

***Corresponding author:** Dr. Woojun Park, Department of Environmental Science and Ecological Engineering, Korea University, Seoul 02841, Republic of Korea

E-mail: wpark@korea.ac.kr

Fax: +82-2-953-0737

Phone: +82-2-3290-3067

Supplementary Table S1. Detailed information of differentially expressed proteins identified by the MALDI-TOF mass spectrometry in the *A. oleivorans* DR1 in the 24h matured biofilms.

Spot no.	Protein	Gene	Locus taq	Start[a]	End[b]	Peptide sequence	Protein sequence coverage (%)[c]	Score[d]	Fold[e]	MW	PI
7707	hypothetical protein		AOLE_07010	84	92	K.LTEDAIREK.K	29	109	261.605 (H)	65.3	6.2
				93	105	K.KTADLGYIQENYK.A					
				106	116	K.ALQQIILLEEK.E					
				206	224	K.KLTEPYAIVVLGGGLTLDK.N					
				281	297	R.SMNTCENTRFSSLLLQK.K					
				298	315	K.KGGAPTVMLVTDEYHMPR.T					
				341	352	R.WQPSIQNYDHSR.R					
				353	363	R.RANYELLATIR.D					
7409	imidazolonepropionase	*hutI*	AOLE_00370	39	54	K.QQQLPTDTYSETVDLK.G	55	167	21	51.8	5.9
				82	103	K.RLQGVSYAEIAASGGGIASTVR.A					
				83	103	R.LQGVSYAEIAASGGGIASTVR.A					
				107	120	R.EASEEQLLNSALKR.I					
				136	146	K.SGYGLNYENER.K					
				136	147	K.SGYGLNYENERK.M					
				154	164	R.QIGEALPMTVK.S					
				225	234	K.TAQSLGLPVK.L					
				235	251	K.LHAEQLSSLGGSSLAAR.Y					
				252	269	R.YHALSADHLEYMTEDDVK.A					
				270	289	K.AMAESGTVAVLLPGAFYLLR.E					
				290	301	R.ETQYPPIESLIK.H					
				306	323	R.IALSSDLNPGTSPALSVR.L					

				324	334	R.LMLNMGSTLFR.L					
				335	365	R.LTPEQALAGVTIHAAQALGLEQTYGSLEQGK.V					
				391	401	K.RVVQHGQEVIF.-					
				392	401	R.VVQHGQEVIF.-					
7806	outer membrane receptor protein, mostly Fe transport	*oprC*	AOLE_18500	139	163	R.ISVIKGPQTVQYANTGSAATVLFER.Q	38	259	10.6	94.1	6
				144	163	K.GPQTVQYANTGSAATVLFER.Q					
				168	187	K.LTSEKPYRGQASVLLGSYGR.I					
				176	187	R.GQASVLLGSYGR.I					
				230	252	K.WNADVALGFTSDEDTWIELTGGK.S					
				230	262	K.WNADVALGFTSDEDTWIELTGGKSDGESLYAGR.S					
				289	309	K.KIEGQVNYSYNDHIMDNFSLR.T					
				290	309	K.IEGQVNYSYNDHIMDNFSLR.T					
				351	363	K.WSLITGVDSQFNK.H					
				386	404	R.FQSYGAFGELGYQWNDLNK.L					
				435	442	K.TLPSAFVR.W					
				443	463	R.WENQHPEHDLKSYIGLGYVER.M					
				454	463	K.SYIGLGYVER.M					
				464	474	R.MPDYWELFSPK.H					
				581	597	K.NTTDDKPLPQISPLEGR.L					
				602	614	R.YVADKYNLGLLWR.A					
				607	614	K.YNLGLLWR.A					
				622	638	R.VSLHQGNIVGYDLKPSK.G					
				639	652	K.GFSTLSLNGSYNLR.K					
				654	675	K.DIDVSVGVDNVLDKTYTEHLNK.A					
				676	694	K.AGSAGFGFASEEQFNNIGR.N					
8012	tRNA-dihydrouridine synthase A	*dusA*	AOLE_16645	1	15	-.MEDKNNWSLYEVFVR.S	86	108	10.1	35.5	6.9
				5	15	K.NNWSLYEVFVR.S					

				24	41	R.HVGSLRAPDDEIALQHAR.D					
				30	41	R.APDDEIALQHAR.D					
				47	57	R.RNEGISIWVVR.S					
				48	57	R.NEGISIWVVR.S					
				58	69	R.SELIKSSQPDEK.A					
				70	82	K.AEFFDPSLDKVYR.H					
				83	96	R.HPTFYHIPDGIEHM.-					
7603	fumarate hydratase	*fumC*	AOLE_07235	5	27	R.IEHDTMGEIEVPNEALWGAQTQR.S	34	146	7.7	59.6	5.8
				28	38	R.SLQNFKIGQER.L					
				117	135	K.LGQVLGAQKPVHPNDHVNR.A					
				186	213	R.THLQDATPLTLGQEFSGYVSQLEHGLVR.L					
				214	244	R.LQQALTGLYELPLGGTAVGTGLNAHPDYAVK.A					
				245	263	K.AAAQLALLTGLPFVTAPNK.F					
				245	270	K.AAAQLALLTGLPFVTAPNKFEALAGR.D					
				436	457	K.QVAVELGLVTAEQFDEVVKPEK.M					
9302	S-adenosylmethionine: 2-DMK methyltransferase and 2-octaprenyl-6-methoxy-1,4-benzoquinone methylase	*ubiE*	AOLE_17670	62	81	R.GAGNTGNVGETTHFGYSTVR.T	40	160	4.1	41.9	7.7
				89	99	K.VAEVFHSVASK.Y					
				100	113	K.YYLMNDLMSFGIHR.L					
				118	126	R.FAINMSGVR.R					
				118	127	R.FAINMSGVRR.G					
				127	143	R.RGQHVLDIAGGTGDLAK.V					
				128	143	R.GQHVLDIAGGTGDLAK.V					
				148	169	R.EVGPQGHVVLSDINESMLNVGR.D					
				148	171	R.EVGPQGHVVLSDINESMLNVGRDR.L					
				209	222	R.NVTDKDAALASMFR.V					
				261	277	K.LVANDSESYKYLAESIR.M					

9601	inosine 5'-monophosphate dehydrogenase	*guaB*	AOLE_00780	37	56	R.GIHLNIPLVSAAMDTVTESR.M	50	191	3.6	62.8	7.5
				73	85	K.NMDIAAQAAEVRR.V					
				89	107	K.FEAGMVKDPITVTPETTVR.E					
				108	125	R.ELIAITTANNISGVPVVK.D					
				129	136	K.VVGIVTGR.D					
				137	157	R.DTRFETNLEQPVSNIMTGQDR.L					
				193	201	K.GLITVTDFR.K					
				193	202	K.GLITVTDFRK.A					
				202	211	R.KAESYPNSCK.D					
				233	259	R.VEALVEAGVDVIVVDTAHGHSAGVIER.V					
				265	295	K.QNFPQVQVIGGNIATGDAALALLDAGADAVK.V					
				329	340	K.DQIPLIADGGIR.F					
				348	378	K.AIGAGASTIMVGSLLAGTEEAPGEVEFFQGR.Y					
				413	421	K.LVPEGIEGR.V					
				441	455	R.SSMGYTGSSVIEDLR.Q					
				441	459	R.SSMGYTGSSVIEDLRQNAK.F					
7802	outer membrane receptor FepA	*fepA*	AOLE_14435	73	84	K.LPVRNDISDYVR.R	38	249	3.3	106.3	5.6
				110	128	R.GMGPENTLILVDGKPINSR.N					
				140	160	R.DTRGDSNWVPAEAIESIEVLR.G					
				143	160	R.GDSNWVPAEAIESIEVLR.G					
				167	181	R.YGSGAAGGVVNIITK.K					
				183	208	K.VTNETHGSLEFYTSQPEDSKEGASNR.V					
				209	219	R.VGFNVSGPLIK.D					
				209	225	R.VGFNVSGPLIKDVLSYR.L					
				263	281	R.LAWQATDQQTVLLDVSSSK.Q					
				282	308	K.QGNIYSGDSQLNANAEADAILSELIGK.E					
				316	330	R.DSYALTHEGDWSWGK.S					

				433	455	R.IASAYIEDNLKLTDSTDAVIGLR.F					
				444	455	K.LTDSTDAVIGLR.F					
				462	475	K.SGSNWSPSLNITQR.L					
				476	483	R.LNDYFTLK.G					
				544	554	K.DIVNASLTWFR.N					
				599	616	K.ALIQGFEGSLGLDFGDIR.W					
				672	697	R.QFAENRLESGIGSGGTNSAIKPSTVK.S					
				698	709	K.SYSTAGINVGYK.I					
				718	726	R.VGVSNLFDK.Q					
5403	acetoin:2,6-dichlorophenolindophenol oxidoreductase subunit beta	*acoB*	AOLE_10255	8	19	R.NAIKEAIESEMR.R	57	230	2.7	48.2	5.1
				20	32	R.RDPTVFVVGEDVR.G					
				21	32	R.DPTVFVVGEDVR.G					
				33	56	R.GGHGGKNTEDNELEGFGGVLGVTK.G					
				39	56	K.NTEDNELEGFGGVLGVTK.G					
				57	66	K.GLWTEFGSER.V					
				159	169	K.VVVPSSPYDVK.G					
				170	177	K.GLLIQAIR.D					
				170	188	K.GLLIQAIRDDDPIVFCEHK.M					
				189	213	K.MLYDIKGEVPDDAYTIPFGVANYTR.E					
				195	213	K.GEVPDDAYTIPFGVANYTR.E					
				214	229	R.EGTDVTIIALGLMVQR.A					
				240	250	K.DGISVEVVDPR.T					
				251	269	R.TISPLDEEGILESVASTGR.V					
				294	299	K.GFHYLK.A					
				300	320	K.APVELVTPPHTPVPFSPVLEK.E					
				300	328	K.APVELVTPPHTPVPFSPVLEKEWIPSVER.I					

				321	328	K.EWIPSVER.I					
3806	TonB dependent receptor family protein	*cirA*	AOLE_02770	36	58	R.IKAHPLEQTSQDFAVADTVVDQK.H	48	345	2.3	101.1	4.8
				93	100	R.GQDGPRVK.V					
				101	128	K.VLQNSSENIDVSTLSPDHAVTVDPVLAK.Q					
				205	222	R.DANNYIAPNYIHEGEKER.R					
				223	244	R.RVDNTFAQGDSVNVGLSWIYDR.G					
				224	244	R.VDNTFAQGDSVNVGLSWIYDR.G					
				245	254	R.GYTGISYSNR.R					
				245	255	R.GYTGISYSNRR.D					
				309	324	R.YDFKTELNDPFAGFQK.L					
				313	324	K.TELNDPFAGFQK.L					
				325	347	K.LRAQASYTDYQHDEIEEGAIATR.F					
				327	347	R.AQASYTDYQHDEIEEGAIATR.F					
				352	379	K.GYDGRVELVHNPIASWEGVIGAQLGQQK.L					
				397	405	K.WSVFALEHK.Q					
				406	417	K.QWKDVHFELSAR.A					
				431	453	K.QDFDGSAFSYAGAANWEFAPNYK.L					
				454	463	K.LSFVASHQER.L					
				536	545	R.LVQYTQDKAR.F					
				546	562	R.FYGAEGEIGYQITPMYK.I					
				563	571	K.ISAFGDYVR.G					
				588	614	R.LGTKVDADFGDGFSGSAEYYHVFNQDK.I					
				592	614	K.VDADFGDGFSGSAEYYHVFNQDK.I					
				650	673	K.ANNLLDDTVYQHASFLSNIPQVGR.N					
				674	683	R.NFTVGVDFSF.-					

[a]Start positions of peptides in matched proteins

[b]End positions of peptides in matched proteins

[c]The percentage of protein sequence coverage (%)

[d]Mascot score from the NCBInr database

[e]Average fold change is the ratio of protein abundance between planktonic cells and biofilms. ‘H’ indicates proteins exclusively detected in biofilm cells

Supplementary Table S2. Detailed information of differentially expressed proteins identified by the MALDI-TOF mass spectrometry in the *A. oleivorans* DR1 in the 24h matured biofilms.

Spot no.	Protein	Gene	Locus taq	Start[a]	End[b]	Peptide sequence	Protein sequence coverage (%)[c]	Score[d]	Fold[e]	MW	pI
102	peroxiredoxin	*ahpC*	AOLE_13380	64	80	K.LGVEIYAVSTDTHFTHK.A	56	220	655.085 (H)	27.8	4.38
				91	106	K.KIQYPLVGDPTWTLSK.N					
				92	106	K.IQYPLVGDPTWTLSK.N					
				107	120	K.NFDVLIESEGLADR.G					
				107	130	K.NFDVLIESEGLADRGTFVIDPEGK.I					
				121	143	R.GTFVIDPEGKIQIVELNAGGIGR.D					
				131	143	K.IQIVELNAGGIGR.D					
				152	169	K.VKAAQYVHAHPGEVCPAK.W					
				154	169	K.AAQYVHAHPGEVCPAK.W					
				170	186	K.WKEGEATLAPSIDLVGK.I					
				172	187	K.EGEATLAPSIDLVGKI.-					
8202	dehydrogenase	*dho*	AOLE_09905	2	12	M.SSTIVVTGAAR.G	51	173	542.275 (H)	34.7	6.75
				21	34	K.KLLQQGYQVIGIDR.Q					
				22	34	K.LLQQGYQVIGIDR.Q					
				35	46	R.QENPEQWEITQK.I					
				47	54	K.IESSEISR.W					
				55	70	R.WQGFQQDITDQETTAK.L					
				101	124	K.TEDWQTLFAVNVMAPIAISQQLAK.H					
				147	156	R.IQLGMYATSK.A					
				165	176	R.NLALEIAPHQVR.L					

204	electron transfer flavoprotein subunit alpha	*etfA*	AOLE_04180	2	27	M.SILVIADHNNQVLNGATLNVVAAAQK.I	69	188	213.89 (H)	37.9	4.27
				28	50	K.IGGDITVLVAGSGAQAVADAAAK.V					
				86	101	K.YVLAASTTTGKNILPR.V					
				125	148	K.RPIYAGNAIATVQSDEAIIVGTVR.G					
				149	171	R.GTAFDPVAAEGGSAAVEAVSDAK.D					
				172	185	K.DAGISHFVSEEIVK.L					
				186	195	K.LDRPELTAAR.I					
				196	212	R.IVVSGGRGVGSGENYHK.V					
				213	220	K.VLDPLADK.L					
				213	229	K.VLDPLADKLGAAQGASR.A					
				230	248	R.AAVDAGFVPNDFQVGQTGK.I					
				249	274	K.IVAPDLYVAVGISGAIQHLAGMKDSK.V					
5605	NAD-dependent aldehyde dehydrogenase	*gabD*	AOLE_06655	17	32	K.SQYENFIGGEWVAPVK.G	47	225	16.2	62.2	5.46
				87	108	K.IADRLEANLEMLAVAETWDNGK.A					
				91	108	R.LEANLEMLAVAETWDNGK.A					
				109	126	K.AVRETLAADLPLAIDHFR.Y					
				112	126	R.ETLAADLPLAIDHFR.Y					
				236	266	K.IAFTGSTQTGQMVMQYATENIIPVTLELGGK.S					
				267	285	K.SPNLFFEDILDKEDDFLEK.T					
				307	324	R.ALVQESIADQFLEMAVER.V					
				330	351	K.TGHPLDTETMIGAQASLQQQEK.I					

				374	388	R.KEVGDGYYVDPTIFK.G					
				375	388	K.EVGDGYYVDPTIFK.G					
				419	434	K.IANDTMYGLGAGVWSR.S					
				442	450	R.AGRAIEAGR.V					
				476	489	R.ENHKMMLDHYQQTK.N					
				490	503	K.NLLVSYSTKPMGFF.-					
1804	TonB-dependent receptor	*fepA*	AOLE_09880	49	69	K.EQDVDKAPASISVITSEEIER.S	57	317	9.4	92.2	4.71
				70	81	R.SAALSLADVLQK.Q					
				82	98	K.QAGVYNYNSGQDKIIIR.G					
				117	125	R.TSSLGAMWR.G					
				126	141	R.GNDFDWSSIPLNSIER.I					
				126	146	R.GNDFDWSSIPLNSIERIEVIR.G					
				147	167	R.GPMSSLYGSDAMGGVINIITK.K					
				168	184	K.KAENGQLHGSVFGQYNR.A					
				169	184	K.AENGQLHGSVFGQYNR.A					
				196	219	R.YGFNLYGGLTDNVSFSLSGDSYNR.D					
				242	265	K.NVHGTLSWDINDQQTLDLDLGYTK.D					
				312	325	K.IYDYDSEYNAPQSR.N					
				326	335	R.NYKQENLLGR.A					
				336	354	R.AFANFDWMMNNTTAGVDYK.D					
				336	357	R.AFANFDWMMNNTTAGVDYKDQK.I					
				373	395	K.SYGVFVQNDTHINDALTLTLGGR.Y					
				410	423	K.AYLAYQLAEGVVLK.G					
				475	495	R.KPNWNAGVTVFENDVENLIER.V					
				496	510	R.VSNPNSPNPTKYPYK.W					
				507	516	K.YPYKWDNVAK.A					

				521	546	K.GVELNGAYDFSDDLGITANATYLDAK.N					
				553	575	K.DLTERPEWLINSSLSWSIVENYR.M					
				593	611	K.ELPAYTTYDVTFTSPLSPR.L					
1904	TonB dependent receptor family protein	*cirA*	AOLE_02770	36	58	R.IKAHPLEQTSQDFAVADTVVDQK.H	61	476	8.7	104	4.68
				38	58	K.AHPLEQTSQDFAVADTVVDQK.H					
				101	128	K.VLQNSSENIDVSTLSPDHAVTVDPVLAK.Q					
				135	156	R.GPSTLLFGAGTVGGLVNVIDNK.I					
				157	173	K.IPTQMPENGYEGQVGLR.Y					
				174	198	R.YNTGSDEKLASAGVTVGLGSQVALR.V					
				182	198	K.LASAGVTVGLGSQVALR.V					
				182	204	K.LASAGVTVGLGSQVALRVEGLTR.D					
				223	244	R.RVDNTFAQGDSVNVGLSWIYDR.G					
				224	244	R.VDNTFAQGDSVNVGLSWIYDR.G					
				245	254	R.GYTGISYSNR.R					
				245	255	R.GYTGISYSNRR.D					
				309	324	R.YDFKTELNDPFAGFQK.L					
				313	324	K.TELNDPFAGFQK.L					
				327	347	R.AQASYTDYQHDEIEEGAIATR.F					
				352	379	K.GYDGRVELVHNPIASWEGVIGAQLGQQK.L					
				357	379	R.VELVHNPIASWEGVIGAQLGQQK.L					
				380	395	K.LNLTGEEAFMAPTTTK.K					
				380	396	K.LNLTGEEAFMAPTTTKK.W					
				396	405	K.KWSVFALEHK.Q					
				397	405	K.WSVFALEHK.Q					

				406	417	K.QWKDVHFELSAR.A					
				409	417	K.DVHFELSAR.A					
				431	453	K.QDFDGSAFSYAGAANWEFAPNYK.L					
				454	463	K.LSFVASHQER.L					
				508	530	K.LDYHLHVYHNWFDDYIYAQTLDR.Y					
				546	562	R.FYGAEGEIGYQITPMYK.I					
				563	571	K.ISAFGDYVR.G					
				592	614	K.VDADFGDGFSGSAEYYHVFNQDK.I					
				615	641	K.IAAYETETEGYNMLNLGVAYSGQYGAK.T					
				650	673	K.ANNLLDDTVYQHASFLSNIPQVGR.N					
				674	683	R.NFTVGVDFSF.-					
3304	malate dehydrogenase	*mdh*	AOLE_02365	7	23	R.VAVTGAAGQIGYSLLFR.I	55	207	2.7	42.9	5.06
				78	94	K.VAFKDADYALLVGSRPR.G					
				82	94	K.DADYALLVGSRPR.G					
				106	124	K.VNGEIFIGQGQALNEVASR.D					
				128	152	K.VLVVGNPANTNAYIAMNSAPDLPAK.N					
				153	159	K.NFTAMLR.L					
				160	172	R.LDHNRALTQVAQK.A					
				173	198	K.AGVAVADIEHLTVWGNHSPTMYADYR.F					
				211	227	K.INDAAWNKDVFLPTVGK.R					
				219	228	K.DVFLPTVGKR.G					
				237	253	R.GLSSAASAANAAIDHMR.D					
				295	306	K.IVQGLEIDEFSR.E					
				309	320	R.INFTLNELEEER.A					

4008	ADP-ribose pyrophosphatase	*nudF*	AOLE_04965	1	16	-.MAAWTPHVTVATVVEK.D	31	99	2.5	22.9	5.2
				2	16	M.AAWTPHVTVATVVEK.D					
				2	19	M.AAWTPHVTVATVVEKDGR.Y					
				124	131	R.ARSPLVLK.A					
				139	162	K.GHHYPLSLIYEHPFSPSLTSHLDA.-					
5709	urocanate hydratase	*hutU*	AOLE_00355	1	9	-.MTTTTTKFR.D	45	296	2.3	74.5	5.42
				8	14	K.FRDVEIR.A					
				15	24	R.APRGTELTAK.S					
				25	33	K.SWLTEAPLR.M					
				49	58	K.ELVVYGGIGR.A					
				75	94	K.NLETDETLLVQSGKPVGVFK.T					
				102	125	R.VLIANSNLVPHWANWEHFNELDAK.A					
				160	169	R.QHYNGDLNGR.W					
				212	226	R.YVDEQATDLDDALAR.I					
				236	254	K.VISIALHGNAAEILPELVR.R					
				236	255	K.VISIALHGNAAEILPELVRR.G					
				307	316	K.HVQAMLDFQK.M					
				317	329	K.MGVPTFDYGNNIR.Q					
				334	357	K.EEGVENAFDFPGFVPAYIRPLFCR.G					
				381	399	K.VKELIPDDEHLHHWLDMAR.E					
				383	399	K.ELIPDDEHLHHWLDMAR.E					
				402	410	R.ISFQGLPAR.I					
				422	431	K.LGLAFNEMVR.S					
				432	443	R.SGELSAPVVIGR.D					
				444	456	R.DHLDSGSVASPNR.E					

				522	533	R.VLTNDPATGVMR.H					
2005	protein pilH	*pilH*	AOLE_03150	4	16	R.ILIVDDSPTETYR.F	81	160	2.1	16	4.88
				17	23	R.FREILTK.H					
				24	42	K.HGYDVIEASNGADGVTLAK.A					
				43	65	K.AEQPDLVLMDVVMPGVNGFQATR.Q					
				70	84	R.DEDTKHIPVVIVSTK.D					
				75	84	K.HIPVVIVSTK.D					
				75	90	K.HIPVVIVSTKDQATDR.V					
				95	109	K.RQGALDYLIKPIEEK.Q					
				96	109	R.QGALDYLIKPIEEK.Q					
2606	F0F1 ATP synthase subunit beta	*atpD*	AOLE_18565	6	20	R.IIQIIGAVIDVEFER.N	51	234	1.9	60.8	4.9
				26	51	K.IYDALQVDGTETTLEVQQQLGDGVVR.T					
				64	85	R.GLNVTSTNAPISVPVGPATLGR.I					
				144	154	K.VGLFGGAGVGK.T					
				168	181	K.AHSGLSVFAGVGER.T					
				218	230	R.VALTGLTMAEYFR.D					
				239	251	K.GRDVLLFVDNIYR.Y					
				241	251	R.DVLLFVDNIYR.Y					
				252	265	R.YTLAGTEVSALLGR.M					
				266	286	R.MPSAVGYQPTLAEEMGVLQER.I					
				348	363	R.QLDPLVVGQEHYEIAR.S					
				364	371	R.SVQNVLQR.Y					
				377	397	K.DIIAILGMDELAEEDKLVVYR.A					
				404	421	R.FFSQPFHVAEVFTGAPGK.L					

				434	460	K.GLLAGEYDHIPEQAFYMVGGIDEVIAK.A					
6410	putative alcohol dehydrogenase	*adh*	AOLE_06670	40	51	K.KALIVTDEGLFK.F	35	241	1.9	51.6	5.85
				41	51	K.ALIVTDEGLFK.F					
				113	125	K.GIGLVTAGGGHIR.D					
				113	132	K.GIGLVTAGGGHIRDYEGIDK.S					
				133	155	K.SKVPMTPLIAVNTTAGTASEMTR.F					
				135	155	K.VPMTPLIAVNTTAGTASEMTR.F					
				168	174	K.MAIVDWR.C					
				168	174	K.MAIVDWR.C + Oxidation (M)					
				226	247	K.AITMISQWLQPAVANGENIEAR.D					
				310	317	K.IADLMGVK.T					
				318	335	K.THGLTVMEAAYAAIDAIR.K					
				318	336	K.THGLTVMEAAYAAIDAIRK.L					
				336	353	R.KLSSSIGIPSGLTELGVK.T					
				337	353	K.LSSSIGIPSGLTELGVK.T					
				376	387	R.KANHAQVVEIFK.A					
				377	387	K.ANHAQVVEIFK.A					
1101	glutathione peroxidase	*gpx*	AOLE_18550	1	11	-.MTQSVYHIPVK.A	60	232	1.8	28.9	4.64
				2	11	M.TQSVYHIPVK.A					
				27	36	K.VLLIVNTASK.C					
				54	69	K.KDQGLEILGFPANNFK.E					
				55	69	K.DQGLEILGFPANNFK.E					
				97	118	K.VSVAGEDKHPLYATLTSAQPER.T					

				105	118	K.HPLYATLTSAQPER.T					
				105	125	K.HPLYATLTSAQPERTGEGPFR.E					
				126	147	R.ERLEGLGIPTNPAPEVLWNFEK.F					
				128	147	R.LEGLGIPTNPAPEVLWNFEK.F					
				153	173	K.NGEVVARFAPNLTADDEQIVK.A					
				160	173	R.FAPNLTADDEQIVK.A					
205	electron transfer flavoprotein subunit alpha	*etfA*	AOLE_04180	28	50	K.IGGDITVLVAGSGAQAVADAAAK.V	65	178	1.7	38.3	4.48
				57	82	K.VLLADNAAYANQLAENVAALVADLAK.G					
				102	124	R.VAALLDVSMITDIISVESANTFK.R					
				125	148	K.RPIYAGNAIATVQSDEAIIVGTVR.G					
				149	171	R.GTAFDPVAAEGGSAAVEAVSDAK.D					
				172	185	K.DAGISHFVSEEIVK.L					
				186	195	K.LDRPELTAAR.I					
				213	229	K.VLDPLADKLGAAQGASR.A					
				230	248	R.AAVDAGFVPNDFQVGQTGK.I					
				249	271	K.IVAPDLYVAVGISGAIQHLAGMK.D					
1006	hypothetical protein		AOLE_15935	2	20	M.TLEYTHKPNYYLFAQLLVR.H	71	154	1.6	16.2	4.68
				21	40	R.HIESYIHKHPDANNAIFDLR.D					
				29	40	K.HPDANNAIFDLR.D					
				41	47	R.DVYEIFR.Q					
				41	68	R.DVYEIFRQDFASTTTNLEGILHIADEYK.I					
				48	68	R.QDFASTTTNLEGILHIADEYK.I					
				88	104	K.NNSLLIDFNSDALTSLR.S					

6504	fumarate hydratase	*fumC*	AOLE_07235	5	27	R.IEHDTMGEIEVPNEALWGAQTQR.S	41	172	1.5	59.3	5.66
				117	135	K.LGQVLGAQKPVHPNDHVNR.A					
				136	167	R.AQSTNDSFPTAIHVAASLQINELLIPAVEQLK.A					
				173	182	K.KSDEFQSIVK.I					
				186	213	R.THLQDATPLTLGQEFSGYVSQLEHGLVR.L					
				245	263	K.AAAQLALLTGLPFVTAPNK.F					
				245	270	K.AAAQLALLTGLPFVTAPNKFEALAGR.D					
				296	302	R.WLASGPR.C					
				397	422	K.IDHFLHNSLMLVTALNPVIGYENSAK.V					
				436	457	K.QVAVELGLVTAEQFDEVVKPEK.M					

[a]Start positions of peptides in matched proteins

[b]End positions of peptides in matched proteins

[c]The percentage of protein sequence coverage (%)

[d]Mascot score from the NCBInr database

[e]Average fold change is the ratio of protein abundance between planktonic cells and biofilms. ‘H’ indicates proteins exclusively detected in biofilm cells

Supplementary Table S3. Bacterial strains, plasmids, and primers used in this study.

Strain/Plasmid/Primer	Description/Sequence (5′-3′) [a]	Source/ Reference
**Strains**		
Acinetobacter oleivorans DR1	Wild type, diesel oil degrader	Jung *et al.* (2010)
ΔoxyR	OxyR (encoded by AOLE_14380) mutant; insertion of pVIK112-*oxyR* into DR1	Lab stock
ΔahpC	AhpC (encoded by AOLE_13380) mutant; insertion of pVIK112-*ahpC* into DR1	Lab stock
ΔsoxR	SoxR (encoded by AOLE_12135) mutant; insertion of pVIK112-*soxR* into DR1	Lab stock
ΔahpC complementation	AhpC (encoded by AOLE_13380) complementation; insertion of pRK415-*ahpC* into *ΔahpC*	This study
A. oleivorans DR1(pRK-P_{ahpC}::*gfp*)	Insertion of pRK415-P_{ahpC}::*gfp* into DR1	This study
Escherichia coli MG1655	Wild type	
LC106	Deletion of *katG*	Linn S & Imlay J. A (1987)
JI361 (HPX)	Deletion of *ahpCF, katG, katE*	Park S, You X & Imlay J.A (2005)
**Plasmids**		
pVIK112	R6K, *oriV*, suicide vector, *lacZ* fusion	Kalogeraki & Winans (1997)
pVIK112-*oxyR*	Km^{r}, internal *oxyR* fragment in pVIK112	Lab stock
pVIK112-*ahpC*	Km^{r}, internal *ahpC* fragment in pVIK112	Lab stock
pRK415	Tc^{r}, Broad-host-range vector	*Yin et al.* (2003)
pRK415-*ahpC* complementation	Tc^{r}, Insertion of the *ahpC* in the pRK415	This study
**Primers**		
***ahpC* mutant**		
ahpC OE-F	CGCCCCGGGTCGAATACAGTGTTTGGGGACT	
ahpC OE-R	CGCGGTACCTGGGTGTGGAATAGAGCTTAGA	
ahpC-F	CGCCCCGGGTGCTTACCACAACGGCCAAT	
ahpC-R	CGCGGTACCCCGATACCACCAGCGTTGAG	

MCS-R	ACCATGGTCATAGCTGTTTCCTG
***ahpC* complementation**	
*ahpC*_comple_F	CGCGGATCCCGCTCTAATGCCCTTCAGGT
*ahpC*_comple_R	CGCGGTACCGCGTGTACGTATTGAGCAGC
M13_FP	TGTAAAACGACGGCCAGT
**P_{ahpC}::*gfp* fusion**	
pRK-P_{ahpC}-F	CGCGAATTCATCTCTGCTGTATTGGCTTTTT
pRK-P_{ahpC}-R	CGCGGTACCGCAGTTGCTTGGAATGGTTTA
pRK::*gfp*-F	CGCGGATCCTGAGATCCTAAAAATCTATCA
pRK::*gfp*-R	CGCGAATTCTTATTTAGCGCTCTTTAATAC
GFP-F	TTGTTGAATTAGATGGCGATGTTA
GFP-R	TTTGGAAAGGGCAGATTGTGT

[a] Underlined bases indicate restriction sites used for cloning

Supplementary Table S4. Primers used to quantify the expression of PNAG genes and amplify the intergenic regions between the PNAG genes and primers used to quantify the expression of *E.coli* EPS.

Target region	Primer	Sequence (5′-3′)	Size (bp)	Source/Reference
**Quantification of gene expression**				
16s rRNA-341F	341F	CCTACGGGAGGCAGCAG		Watanabe *et al*.
16s rRNA-534R	534R	ATTACCGCGGCTGCTGGCA		Watanabe *et al*.
AOLE_14655	PNAG_14655_qRT PCR_F	TTTGGTTGTTGTTGTGGCGT	174	This study
	PNAG_14655_qRT PCR_R	GCTCAGGTAGCAAATGGCTC		This study
**Intergenic regions**				
AOLE_14650..AOLE_14655	Int_14650..14655_F	GCGATATTGATGTACCGTGTCA	200	This study
	Int_14650..14655_R	AGCAGGGACGTAGCCAAAAA		This study
AOLE_14655..AOLE_14660	Int_14655..14660_F	CCACATCATGGCTGTGCTTG	225	This study
	Int_14655..14660_R	TGAGAAACGAGCACGTTGGG		This study
AOLE_14660..AOLE_14665	Int_14660..14665_F	TGATCGCACCCATCATCCAT	174	This study
	Int_14660..14665_R	AGATCACCCGGATGTTGACG		This study
AOLE_14665..AOLE_14670	Int_14665..14670_F	CCAAGGCCAAGCCAAGTGAT	388	This study
	Int_14665..14670_R	ATGGCGAGCGTAAAATGGGT		This study
AOLE_14670..AOLE_14675	Int_14670..14675_F	ACTGCTTCTTTGCCTTCACC	179	This study
	Int_14670..14675_R	GGTCAGGCAAATGTTGTTTGGA		This study
AOLE_14675..AOLE_14680	Int_14675..14680_F	TGGCAATATTCAAGTCTGCCT	308	This study
	Int_14675..14680_R	GCCCCAACTGCAACTCCTAA		This study
***E.coli* EPS expression**				
rfbD (b2040) ex-F		CTGGCAAAAGAGCGTGAAGAAT		

rfbD (b2040) ex-R	AAAAACCAGCGCAGCATAATCGTA
pgaC (b1022) ex-F	TGGCGGCGTCTATTTCTGGGTCTA
pgaC (b1022) ex-R	TGTGCTAAAGCGGCGTGTATGGTT
wzc (b2060) ex-F	CCAGCCCGGGCAGTGAGTTT
wzc (b2060) ex-R	ACGGGCGATGCTGTTAAGAATGTC
yjbH (b4029) ex-F	TCGGCCTGGGCTGGGGGTATT
yjbH (b4029) ex-R	GGTATTCCACGCCGCCAAACAGT

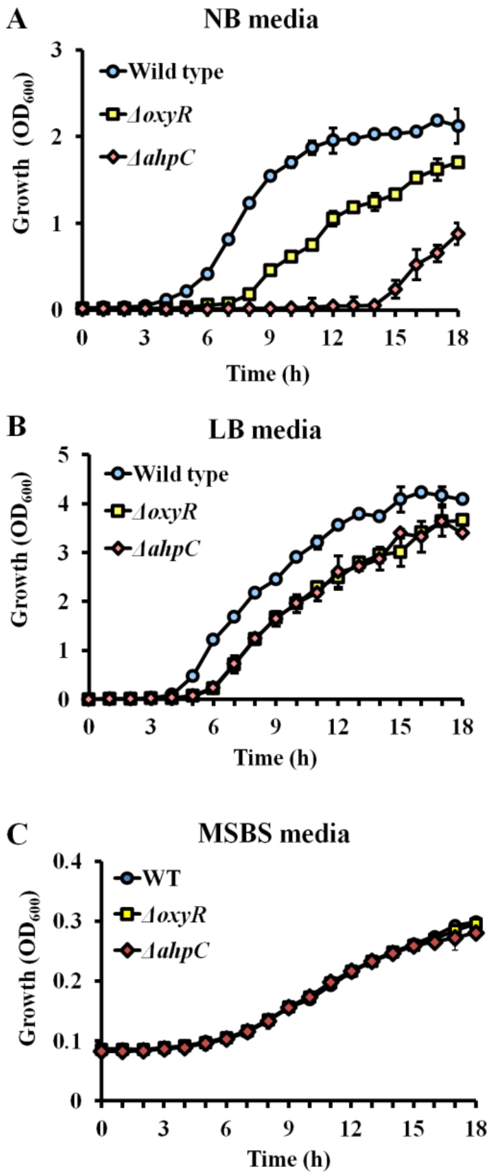

Supplementary Fig. S1. Growth measurements in different culture media. (A) Growth was monitored in LB medium at 30 ℃. (B) Growth was monitored in NB medium at 30 ℃. (C) Growth was monitored in MSBS medium at 30 ℃.

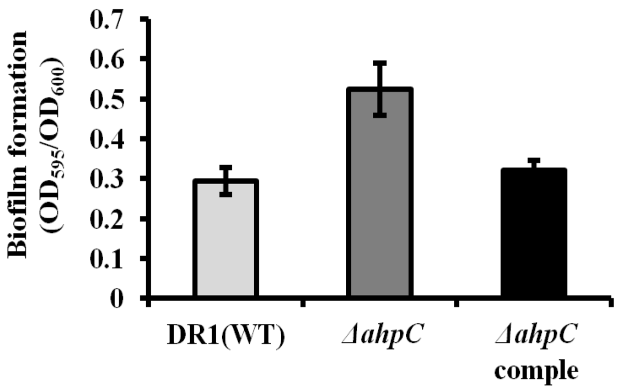

Supplementary Fig. S2. Recovery of biofilm formation in the *ahpC* complementation strain. Comparison of biofilm formation in DR1 (WT), the a*hpC* mutant and the *ahpC* complementation strain (comple).

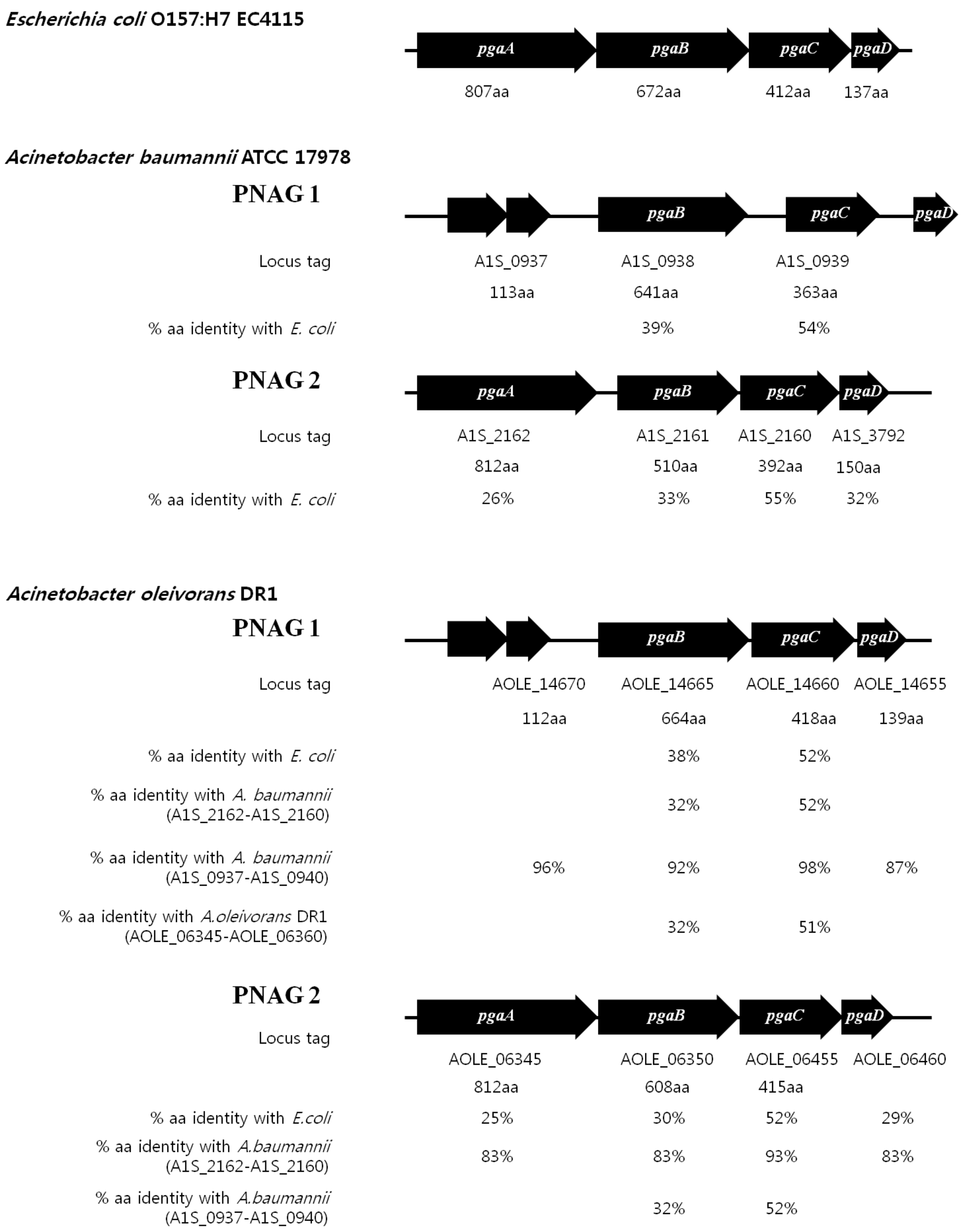

Supplementary Fig. S3. PNAG homologs in *A. oleivorans* DR1, *E. coli*, and *A. baumannii*. *A. oleivorans* DR1 has two PNAG operons. One is located from nucleotides 3,132,165 to 3,137,139 and the other one is located from nucleotides 1,382,343 to 1,389,531. The percent identity is shown below each operon.

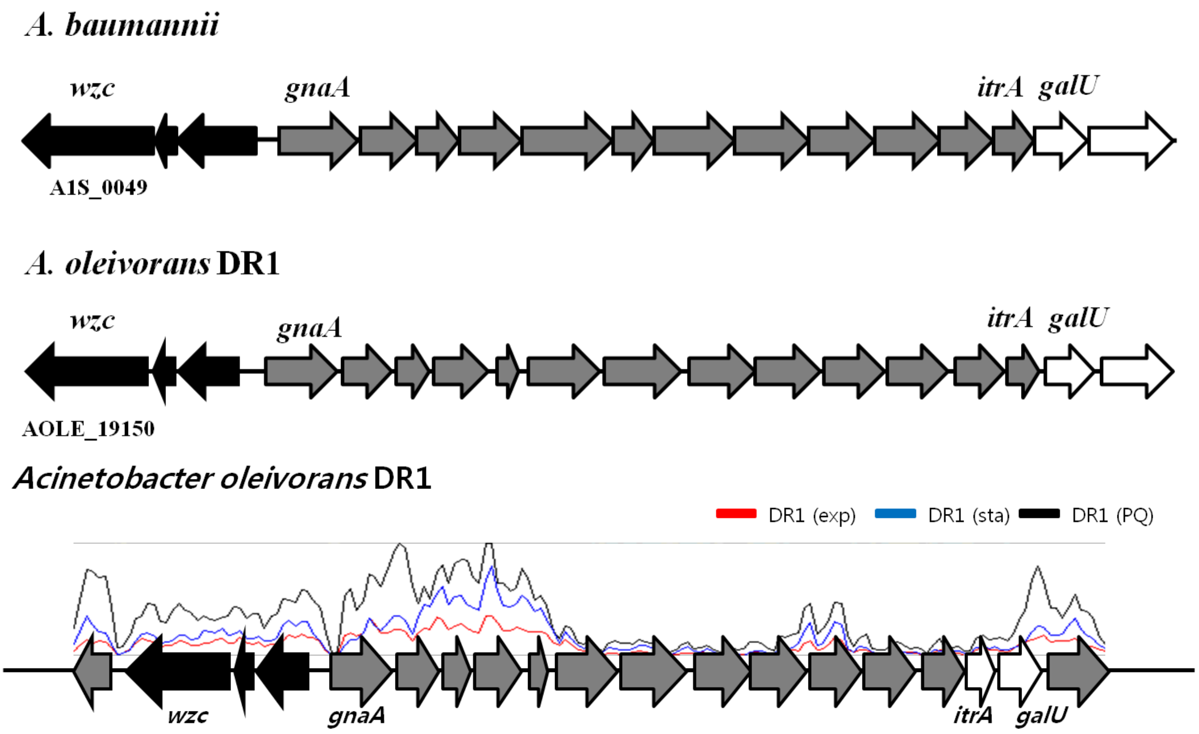

Supplementary Fig. S4. K locus homologs in *A. oleivorans* DR1 and *A. baumannii*. The K locus, which encodes proteins involved in the production of complex polysaccharides, in *A. oleivorans* DR1 includes 19 genes. The K locus operon induced under oxidative stress.

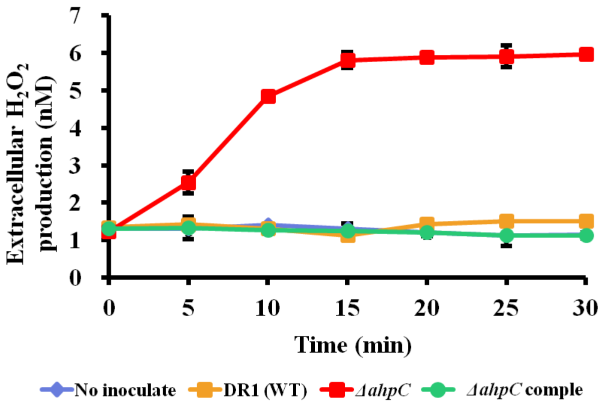

Supplementary Fig. S5. Recovery of H_2O_2 removal in the *ahpC* complementation strain. H_2O_2 production in DR1 (WT), the *ahpC* mutant and the *ahpC* complementation strain (comple).

E. coli	*pgaC*	129	YLVCI**D**GDALL	-----	222	DM I TE**D**IDISW
A. baumannii	*pgaC* 1	80	FLI GI**D**GDALL	-----	173	NMLTE**D**IDITW
	pgaC 2	107	YLVCI**D**GDALL	-----	200	D K I TE**D**IDISW
A. oleivorans DR1	*pgaC* 1	136	FLI GI**D**GDALL	-----	229	NMLTE**D**IDITW
	pgaC 2	130	YLVCI**D**GDALL	-----	223	D K I TE**D**IDISW

E. coli	*pgaC*	258	KGLWK**Q**RL**RW**AQGGA
A. baumannii	*pgaC* 1	209	NGLWK**Q**RL**RW**AMGGA
	pgaC 2	236	NGLWK**Q**RL**RW**AMGGA
A. oleivorans DR1	*pgaC* 1	265	NGLWK**Q**RL**RW**AMGGA
	pgaC 2	259	KGLWK**Q**RL**RW**AQGGV

Supplementary Fig. S6. Partial alignment of *A. oleivorans* DR1 *pgaC* with other glycosyltransferases. The predicted PgaC protein sequence (418 aa) was compared with PgaC sequences from *E. coli* and *A. baumannii.* Alignment of conserved amino acids was generated by CLUSTAL W in the Lasergene MegAlign program. ECH74115_1264 (412 aa) and *A. baumannii* ATCC 17978 A1S_0939 are shown as PgaC1, and A1S_2160 is shown as PgaC2. The regions containing the five amino acids that are predicted to function as catalytic residues are shown. The conserved amino acids (shown in red) are Asp^{141}, Asp^{234}, Gln^{270}, Arg^{273}, and Trp^{274} in *A. oleivorans* DR1 PgaC1 and Asp^{135}, Asp^{228}, Gln^{264}, Arg^{267}, and Trp^{268} in PgaC2.

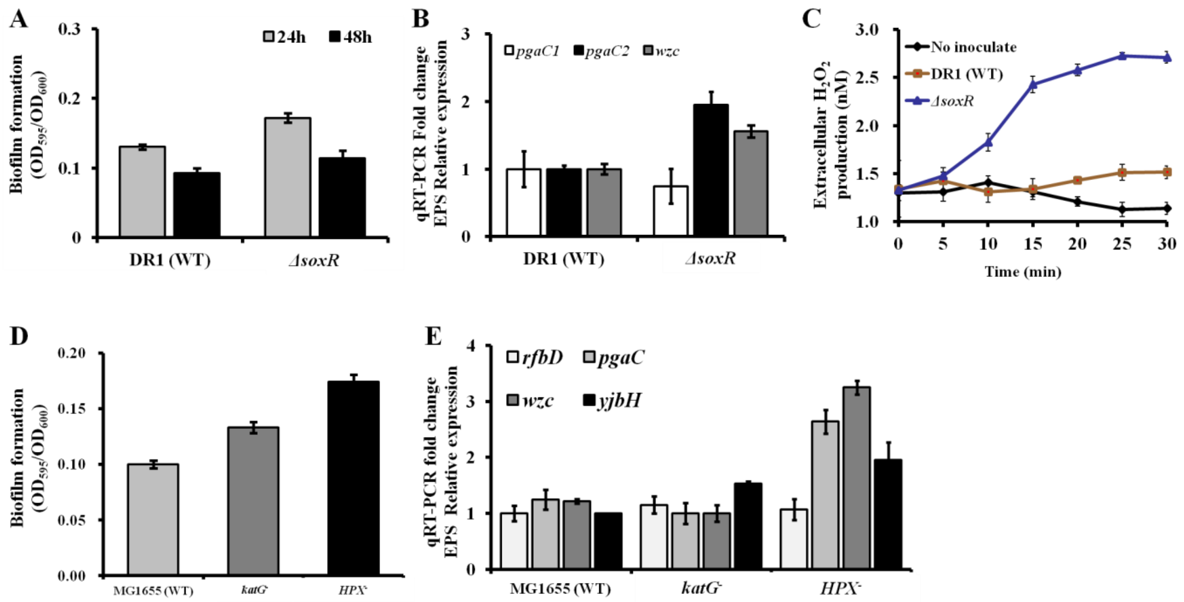

Supplementary Fig. S7. Endogenous oxidative stress enhanced biofilm formation by inducing different EPS operons. (A) Biofilm formation in the *A. oleivorans* DR1 *soxR* deletion mutant during 24h and 48h. (B) Expression of three different EPS biosynthesis operons in the *soxR* mutant. (C) H_2O_2 production in the *soxR* mutant. (D) Biofilm formation in *E. coli* MG1655 (WT), the *katG*⁻ and Hpx mutants. (E) Expression of four different EPS biosynthesis operons in MG1655 (WT), the *katG*⁻ and Hpx mutants⁻.
